# Modulation of the Berry Skin Transcriptome of cv. Tempranillo Induced by Water Stress Levels

**DOI:** 10.3390/plants12091778

**Published:** 2023-04-26

**Authors:** Luísa C. Carvalho, Miguel J. N. Ramos, David Faísca-Silva, Pedro Marreiros, João C. Fernandes, Ricardo Egipto, Carlos M. Lopes, Sara Amâncio

**Affiliations:** 1 Linking Landscape, Environment, Agriculture and Food Research Centre (LEAF), Associated Laboratory TERRA, Instituto Superior de Agronomia, Universidade de Lisboa, 1649-004 Lisboa, Portugal; miguelramos22@gmail.com (M.J.N.R.); davidfaiscadasilva@gmail.com (D.F.-S.); pedromarreiros2009@gmail.com (P.M.); jcfernandes@isa.ulisboa.pt (J.C.F.); carlosmlopes@isa.ulisboa.pt (C.M.L.); 2 INIAV—Instituto Nacional de Investigação Agrária e Veterinária, Polo de Inovação de Dois Portos, 2565-191 Dois Portos, Portugal; ricardo.egipto@iniav.pt

**Keywords:** berry skin, deficit irrigation strategies, RNA-Seq, sHSP, ethylene, auxins

## Abstract

Climate change in the Mediterranean area is making summers warmer and dryer. Grapevine (*Vitis vinifera* L.) is mostly important for wine production in Mediterranean countries, and the variety Tempranillo is one of the most cultivated in Spain and Portugal. Drought decreases yield and quality and causes important economic losses. As full irrigation has negative effects on quality and water is scarce in this region, deficit irrigation is often applied. In this research, we studied the effects of two deficit irrigation treatments, Sustained Deficit Irrigation (SDI) and Regulated Deficit Irrigation (RDI), on the transcriptome of grape berries at full maturation, through RNAseq. The expression of differentially regulated genes (DEGs) was also monitored through RT-qPCR along berry development. Most transcripts were regulated by water stress, with a similar distribution of up- and down-regulated transcripts within functional categories (FC). Primary metabolism was the more severely affected FC under water stress, followed by signaling and transport. Almost all DEGs monitored were significantly up-regulated by severe water stress at veraison. The modulation of an auxin response repression factor, *AUX22D*, by water stress indicates a role of this gene in the response to drought. Further, the expression of *WRKY40*, a TF that regulates anthocyanin biosynthesis, may be responsible for changes in grape quality under severe water stress.

## 1. Introduction

Abiotic stresses are a foremost restricting factor impairing grapevine growth, yield, productivity, and long term survival in traditional Mediterranean climate areas and in new production regions. Drought is one of the most critical environmental factors affecting plant water uptake, and short and long-term adaptation of grapevine varieties to climate change [1].

In regions with hot and dry summer seasons, major adaptations of plant metabolism are required to cope with those adverse environmental conditions. Such adaptations include regulation of transcription and gene expression, and wide transcriptome reprogramming as a response to the stress [2]. Therefore, transcriptomic studies are of particular relevance to understand the mechanisms of plant stress responses. Plant hormones play an important role in the modulation of the complex plant physiological and molecular responses to drought, coordinating water loss and the maintenance of cellular growth, as is the case of abscisic acid [3]. However, this is only one among the many key players in the complex molecular networks that lead to the inhibition/induction of key proteins in stress signal reception, transmission, and responses, such as kinases, phosphatases, transcription factors, and defense response genes [4,5,6]. Some key transcription factor (TF) families such as MYB, WRKY, and bZIPs have been found to be involved in various processes depending on the type of stress. In fact, several TFs have been object of genetic engineering to improve stress tolerance in some species of interest (for review, see [7]).

Grapevine is one of the most important crops worldwide and the wine industry is one of the most globalized and competitive. Impending global climate change stands to severely impact the wine industry and the economy of southern Europe, through decreases in yield and grape quality [8,9]. Episodes of intense drought associated with heat increase the demand for evaporative cooling, thus accelerating the loss of water. This impairs the long-term survival of the plant. Furthermore, higher temperatures and intense droughts during the season anticipate phenology by several days and also, when occurring close to berry maturation, they increase the alcohol content of the berry, changing the wine’s sensory profiles [10]. During ripening, sugar accumulation increases with temperature but secondary metabolites, such as anthocyanins, are negatively affected by high temperature while berry acidity, in particular malic acid content, decreases [11]. A comprehensive understanding of the mechanisms of response of grape berries to limited water availability is a prerequisite to the advance of breeding and cultural practices necessary for enhancing grapevine tolerance to limited water availability, and to implement sustainable deficit irrigation plans. Each grapevine variety has a unique response to stress [12], that can be characterized at the clone level [13].

With that challenge in mind, we set out to comprehend the impact of different levels of deficit irrigation (Sustainable Deficit Irrigation and Regulated Deficit Irrigation) on the berry transcriptome of the widely cultivated grapevine variety Tempranillo. The modulation of expression of key transcripts related to the drought-induced defense mechanisms were also monitored in key phenological stages, to characterize its response to stress.

## 2. Results

**Figure 1:**
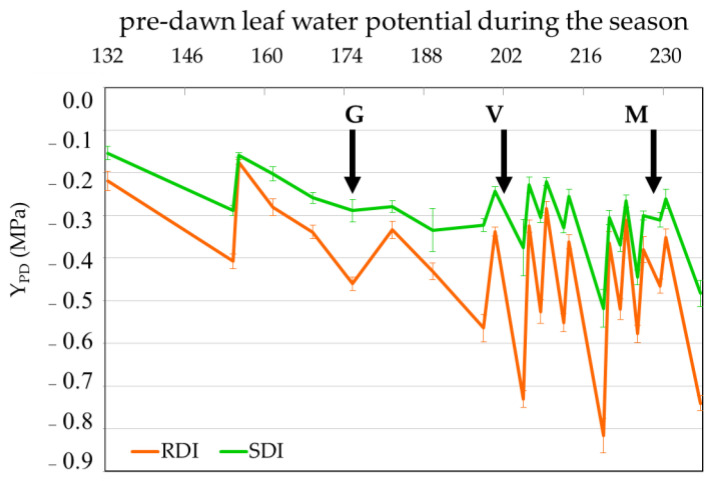
Pre-dawn leaf water potential (Ψpd) during the season (day of the year) in Sustained Deficit Irrigation (SDI) and regulated Deficit Irrigation (RDI), plants at Esporão vineyard (38°23′42.0″ N 7°32′51.4″ W: Sampling at green pea (G), veraison (V) and full maturation (M) indicated with black arrows.

The values of pre-dawn leaf water potential along the season in both deficit irrigation treatments (Sustainable Deficit Irrigation, SDI, and Regulated Deficit Irrigation, RDI) are depicted in Figure 1. A pattern of severe water stress was observed in RDI, with Ψ_PD_ reaching values lower than −0.7 MPa on several days between veraison and full maturation.

### 2.1. Overall Transcriptomic Response to Water Deficit

**Figure 2:**
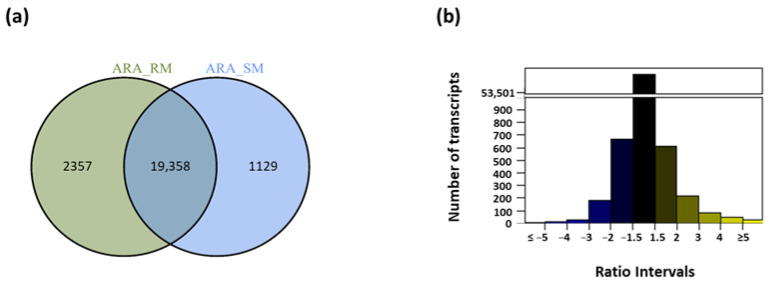
(**a**) Venn diagram showing sample-specific transcript analysis considering only the transcripts with length ≥ 150 bp and a threshold of RPKM ≥ 1 for a transcript to be considered as active in full maturation samples under Regulated deficit irrigation (RM) and under Sustained deficit irrigation (SM); (**b**) Number of transcripts distribution per ratio interval (RDI/SDI) in Tempranillo full maturation samples. A scale break was applied from ≈1000 and ≈50,000 transcripts.

To determine the number of transcripts exclusive to each irrigation strategy, a Venn diagram was built. At full maturation (M), 22,844 transcripts were identified, in RDI and SDI (Figure 2a). A total of 1129 transcripts were exclusively expressed in SDI, less than half the number of those expressed in RDI, 2357. This is an indication that the irrigation strategies under study induced major changes in transcript modulation in water stressed plants. Therefore, to appraise which transcripts were differentially regulated by each irrigation strategy, the expression ratio was calculated, considering SDI as control (Table S2). To visualize the transcript ratio distribution, a barplot was made with ratio intervals of RDI/SDI. Most transcripts show a ratio between −1.5 and 1.5 (Figure 2b), an indication that they have similar expression on both irrigation strategies, demonstrating that they were not regulated by the level of water deficit. A few transcripts (approximately 30) had their expression ratio on the extreme sets (ratio ≤ −5 or ratio ≥ 5), showing significant response to water stress.

**Figure 3:**
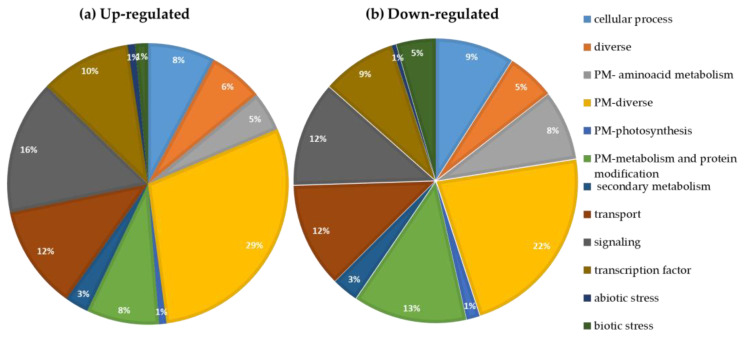
Functional category analysis of significantly regulated DEGs in Regulated deficit irrigation (RDI) relative to Sustained deficit irrigation (SDI). The values of gene expression used for the analysis were obtained in the RNA-seq analysis and all the transcripts represented in the figure showed significant differences in gene expression between SDI and RDI at full maturation (RDI/SDI ratio < 0.25: down-, and >4: up-). (**a**) Up-regulated DEGs; (**b**) Down-regulated DEGs. PM: Primary Metabolism.

The ratio RDI/SDI reflects a differential regulation of expression in genes belonging to various functional categories that is modulated by water stress. The percentage of genes up- and down-regulated per functional category was plotted and is shown in Figure 3. Comparing the main functional categories there are similarities between the percentages of up- and down-regulated transcripts. However, it is possible to mention that the categories, cell process, PM (Primary Metabolism)—amino acids metabolism, PM-protein, and biotic stress have a higher percentage of down-regulated transcripts (between 15 and 40%, depending on the category) than up-regulated transcripts. The reverse occurred in the categories PM-miscellaneous, signaling and transcription factors, although the differences did not exceed 20–30%. Overall, the categories PM-photosynthesis and response to abiotic and biotic stress are the least represented, with values below 2% in up- and between 0.5 and 4.5% in down-regulated.

The functional categories signaling and transcription factors were analyzed in more detail (Table 1). In the signaling category, DEGs (differentially expressed genes) were distributed in nine subcategories. Kinases was the most represented subcategory, with up- and down-regulated DEGs, while members of the calcium sensor subcategory were only up-regulated. As for the hormone related DEGs, they presented a greater number of up-regulated transcripts, in particular auxins and cytokinins. Transcription factors had twelve subcategories, the most represented was zinc finger (with up- and down-regulation) while MYB transcription factors were only up-regulated.

### 2.2. Modulation of Expression of Selected Differentially Expressed Genes (DEGs)

**Figure 4:**
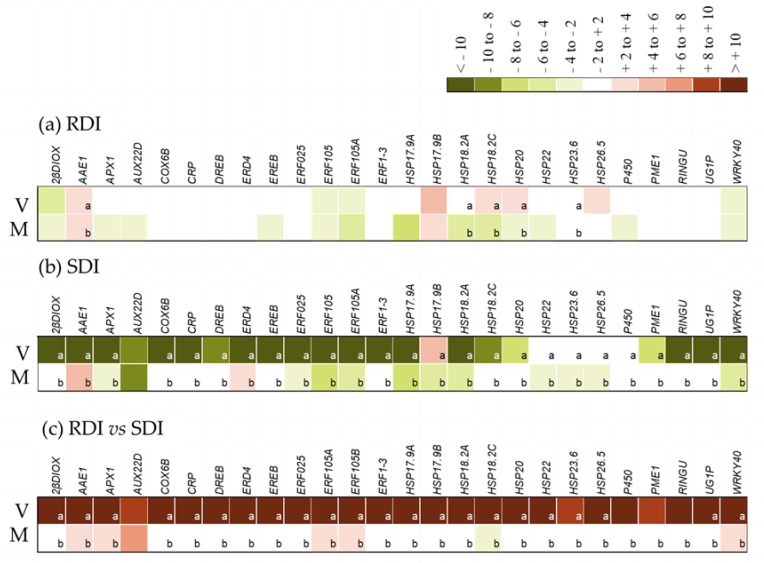
Heat map representing gene expression ratios quantified by RT-qPCR of the DEGs of interest (*2βDIOX, AAE1, APX1, AUX22D, COX6B, CRP, DREB, ERD4, EREB, ERF025, ERF105A, ERF105B, ERF1-3; HSP17.9A, HSP17.9B, HSP18.2A, HSP18.2B, HSP20, HSP22, HSP23.6, HSP26.5, P450, PME1, RINGU, UG1P, WRKY40*) in (**a**) sustained deficit irrigation (SDI) in veraison (V), and full maturation (M), when using the phenological stage green pea, G, as control; in (**b**) regulated deficit irrigation (RDI) in V and M, also using G as control; and in (**c**) RDI compared to SDI (as control), in V and M. RT-qPCR values were standardized with the CT values of the reference genes *Actin 2; TIF*, and *TIF-GTP*. Different letters indicate significant differences (*p*-value < 0.05) in the expression of each DEG between the development stages. Values within |log2(gene expression level)| < 2 are not significantly different from the respective controls (G vs. V and M in (**a**,**b**); RDI vs. SDI in (**c**)).

The ten genes with the highest and lowest levels of expression are shown on Table 2. Most of them are members of the Metabolism Functional Category. The gene with the highest expression ratio is VIT_217s0053g00010.1, a non-annotated transcript, with no similarities to any expressed sequence. The gene with the second highest ratio (VIT_209s0018g00240.2) is a WRKY transcription factor, thus within the Transcription factor Functional Category. As for the most down-regulated gene (VIT_212s0035g01900.3), it encodes a pectin methylesterase, involved in cellular processes. As most of those ten DEGs were still non annotated and some of the annotated ones belong to the primary metabolism category, well known to be down-regulated upon stress, we opted to choose the DEGs for qPCR analysis within the whole list of significantly up-and down-regulated genes and not just from the restricted number shown in Table 2. Therefore, 26 DEGs were chosen, within stress responsive functional categories (Table S3 for names, primer sequences and expression levels in the RNAseq experiment). Their relative expression was evaluated through RT-qPCR, with two different comparisons. The expression of RDI and SDI transcripts was compared at veraison and full maturation, using green pea (G) as control, and the expression between RDI and SDI transcripts was compared at veraison and full maturation (Figure 4).

The analysis of expression in RDI throughout berry development (Figure 4a) showed medium levels of upregulation of four *sHSPs* and *AAE1*, while two *ERFs*, *WRKY40* and *2βDIOX* were down-regulated at veraison, when compared to green pea stage. At full maturation, only *AAE1* and *HSP17.9B* were significantly up-regulated. In SDI, all DEGs were significantly down-regulated at veraison, and most kept a down-regulation pattern through maturation, except for AAE1 and ERD4, significantly up-regulated (Figure 4b). At veraison, the comparison of RDI with SDI revealed significant up-regulation of all the DEGs, only a few of which were still up-regulated at full maturation (*AAE1, APX1, AUX22B, ERF105A, ERF105B* and *WRKY40*). The only significantly down-regulated DEG at full maturation in RDI was *HSP18.2C* (Figure 4c).

## 3. Discussion

The main objective of this project was to monitor the expression of transcripts of interest in the berry skins of Tempranillo in three stages of development (G, green pea; V, veraison; M, full maturation) under two different deficit irrigation conditions, SDI (control, mild water stress) and RDI (severe water stress). To achieve this goal, global gene expression was obtained by RNA-Seq at full maturation and target gene expression was monitored in the three developmental stages using RT-qPCR.

In a global first analysis, a common feature of the distribution graphs of the transcripts into functional classes in SDI and RDI is the fact that the Primary Metabolism category represents the highest percentage of the graph, occupying about 50%. One of the main explanations for this result is the fact that the Primary Metabolism category involves many processes of extreme importance for the survival of the plant, such as the metabolism of carbohydrates, lipids, nitrogen, sulfur, carbon fixation, among others. Of these processes, carbohydrate metabolism tends be down-regulated, since the decrease in basic metabolism processes is typical in stress conditions [14,15], as those of RDI.

The response to water stress, as to any other type of stress factor, is mediated by plant hormones [16,17]. A typical response to water stress is the synthesis of abscisic acid (ABA), which is transported to the shoots, inducing changes in the expression of genes related to the plant’s response to drought [18,19]. ABA synthesis also promotes stomatal closure, which reduces water loss through transpiration [20]. In the current RNA-seq study, there were more up-regulated transcripts of the ABA category than down-regulated. Ethylene also takes part in the response to stress by strengthening the antioxidant machinery and by interacting with other signaling molecules to trigger a cascade of adaptive responses [21], as confirmed by the results of *ERF* gene expression. In the case of auxins, when under severe water stress (RDI), the plant activated genes within this category, which differs from the results reported by Zhang et al. [22], and by Carvalho et al. [23], where the inhibition of the auxin pathway enabled the plants to withstand drought. In fact, the decrease in IAA can provide tolerance by facilitating the action of ABA [20]. However, in the present work, ABA and auxins appear to work synergistically, a response that may be variety-dependent, as Touriga Nacional grown in similar experimental conditions, showed an inhibition of the auxin pathway [23].

Our results also show that there is a high number of transcripts encoding kinases that are activated when the plant is under severe water stress, certainly because these proteins are the main components of intracellular signaling and are responsible for rapid responses to changes in the environment [24]. In plants there are kinases linked to several processes of development and control of primary and secondary metabolism, including photosynthesis and anthocyanin biosynthesis [25,26,27]. Abiotic stress induces the transcription of molecules that respond to calcium [28], which also occurred in the present study.

As transcription factors are mediators between the appraisal of environmental signals and the expression of stress response genes, they act as switches of regulatory transcription cascades enabling the adaptation of plants to environmental changes [29,30]. In the present RNA-seq analysis, *MYB* and *Zinc Finger* families had several up-regulated genes in RDI, which is in line with the literature [31,32]. Studies focused on gene expression have shown that genes in the Zinc Finger family, such as *C2H2* and *C3HC3*, are induced by several types of abiotic stress, including drought [33,34]. The MYB family has also been described in the regulation of responses to abiotic and biotic stresses [35], with elements such as *AtMYB88*, which increased the tolerance to abiotic stress when overexpressed in Arabidopsis, restricting the divisions that occur at the end of the stomatal cell line [36].

In a more detailed analysis, using RT-qPCR, two main families of genes were studied in detail, those coding for Heat shock proteins (HSP) and Ethylene Responsive Factors (ERF), along with other genes that are also associated with responses to abiotic stress. In Tempranillo at veraison, all the genes studied had significantly higher levels of expression in RDI than in SDI, while at full maturation, the differences were atoned. This result differs from the one obtained in Touriga Nacional under equivalent experimental conditions [23], and where the peak of gene expression occurred at full maturation. Tempranillo’s response to water deficit was also studied in leaves, and an overall decrease in gene expression was observed, accompanied by punctual increases in genes necessary for survival [37].

During the first two weeks of berry development, its size increases markedly, as auxins directly promote cell division and growth [38,39]. However, it has been documented that auxins have a negative role during grape ripening [40], which, in the current work, was true for the treatment with low levels of stress. The transcript coding AUX22D, an auxin response repression factor which forms heterodimers with ARFs (auxin response factors), is described as increasing with berry development [41]. Unlike that report, in SDI and RDI, *AUX22D* significantly decreased throughout berry development. This decrease was modulated by water stress level, with the control decreasing to significantly lower levels than the severe water stress.

The HSP family responds to a wide range of abiotic stresses [42], conferring tolerance by preserving the integrity of cell membranes and proteins, and it is known that after the stressor factor ends, they return to baseline levels [43]. In grapevine berries several *HSP* are predominantly induced during ripening [44,45] due to berry dehydration [46], but they can also be present at other stages of development [47]. For these reasons, it was expected that the *HSPs* analyzed would have increased their expression in RDI at full maturation, as they did at veraison, but in fact, they decreased to levels significantly lower than before the application of stress (at the green pea stage).

The ERF family TFs confer plants’ tolerance to stress through different mechanisms, such as inducing the expression of genes associated with resistance to stress [48], hormonal cross-talk [49], and genes involved in the response to reactive oxygen species (ROS) [50,51,52,53]. Therefore, it was expected that the *ERF* family genes studied would be up-regulated during water stress. However, the *ERFs* studied accumulated significantly in response to drought only at veraison. At full maturation, only two isoforms of *ERF105* were significantly up-regulated by drought. These intriguing profiles suggest that different ethylene signaling pathways can be induced at different phases of berry development, possibly also in response to stress [54]. In fact, they could play a role in the regulation of *WRKY40*, which had the same pattern of expression as both isoforms of *ERF105*. In fact, in *Vitis amuriensis*, one such relation was described in the response to cold stress, where *VaERF092* regulates the expression of *VaWRKY33* [55]. *WRKY40* is described as controlling anthocyanin biosynthesis in red apple [56] and could be responsible for changes in grape quality under severe drought stress.

## 4. Materials and Methods

### 4.1. Field Conditions and Sampling

Berry samples were collected during the growing season in grapevine plants of the variety Tempranillo, in an experimental field located at Herdade do Esporão, Reguengos de Monsaraz (38°23′42.0″ N,7°32′51.4″ W). The plants were subjected to two irrigation strategies, Sustainable Deficit Irrigation (SDI), corresponding to 36% potential crop Evapotranspiration (ETc), as the control, and Regulated Deficit Irrigation (RDI), the deficit irrigation treatment, at 24% ETc. Water stress levels were monitored regularly along the season (Figure 1) through measurements of pre-dawn leaf water potential (ψPD), using a pressure chamber, Model 600, PMS Instruments Company (Albany, OR, USA). Berries were collected at three key phenological stages: green pea (BBCH stage 75, G), veraison (BBCH stage 81, V), and full maturation (BBCH stage 85–89, M). Three samples from both irrigation strategies and the three berry development stages were composed of clusters from ten plants each, comprising three berries from the top, three from the middle, and three from the bottom sections of one cluster per plant. The berries were transported on ice to the lab where the skins of 40 berries per sample were removed, reduced to powder in liquid nitrogen and kept at −80 °C until further analysis. RNA-seq was performed on two replicates of M samples while RT-qPCR was performed on three replicates of samples collected at the three phenological stages.

### 4.2. RNA Extraction for RNA Seq and for RTqPCR

RNA extraction was performed with Spectrum Plant Total RNA kit (Sigma–Aldrich, St. Louis, USA), according to the manufacturer’s instructions. After the extraction, RNA samples were treated with RNase-free DNase I (Qiagen, Hilden, Germany) according to the manufacturer’s protocol. RNA was quantified through spectrophotometry in a Synergy HT Multiplate Reader (BioTek, Friedrichshall, Germany) with a Take3 Multi-Volume Plate (BioTek), with Gene5 software (BioTek). Sample quality and integrity were assessed through the A260/A280 ratio and through visual analysis on 2% agarose electrophoresis, respectively. Only samples with a ratio between 1.8 and 2.1 and with the two ribosomal RNA bands clearly visible were used for RNA-sequencing (RNA-seq).

### 4.3. Transcriptome Sequencing and Mapping, and Gene Expression

Sequencing was performed in the Genomics and Transcriptomics Platform, University of Torino, Italy (https://cpt.univr.it/en/genomics-and-transcriptomics-platform/, accessed on the 14 September 2016), as described in Carvalho et al. [23]. Read counts of sequencing, fragment length, and GC percentage of fragments before and after trimming are shown on Table S1.

In all samples, the number of Reads per Kilobase transcript per Million reads (RPKM) was individually calculated, and it matched the cleaned reads library from each sample to the reference transcriptome (versions 12X_v2.1 of *Vitis vinifera vinifera* cv. PN40024, downloaded from Grape Genome Database on CRIBI (https://genomes.cribi.unipd.it/grape/, accessed on the 14 September 2016), as described in Carvalho et al. [23].

### 4.4. Bioinformatics Analysis, Validation and Overview

RStudio R i386 3.3.2 [57] was used for the analyses, with the script used by Carvalho et al. [23]. The function cor (method = “Pearson”, use = “pairwise.complete.obs”) was used for bioinformatics validation. Natural logarithm was applied to the RPKMs obtained and the correlations were assessed. Plots for all samples were drawn using the function ggplot from the package ggplot2 (2.2.1) [58] and are shown on Supplemental Figure S1. A linear regression was traced using the function geo_smooth, from ggplot2. Since both correlations (coefficient of determination 0.94 for SDI and 0.88 for RDI; *p*-value < 2.2 × 10^−16^) were strong, the data contained in the replicates were merged and the average of the replicates was used for further analyses.

### 4.5. Differentially Expressed Genes

In all transcripts the value of RPKM in the RDI treatment was divided by the RPKM measured in SDI (here used as the control) to assess how the expression levels of the transcripts changed in RDI relatively to SDI (Table S2). A cut off value of 1.5 was considered for significance, as in Carvalho et al. [23]. The differentially expressed genes (DEGs) were then ordered by their RDI/SDI values and the ten most up-regulated and the ten most down-regulated were used for further bioinformatics studies. The sequences were identified from the Grape Genome Database on CRIBI (https://genomes.cribi.unipd.it/grape/, accessed on the 14 September 2016) by matching the transcript ID, and submitted to a blastn [59] on TAIR, the *Arabidopsis Thaliana* Genome Database (https://www.arabidopsis.org, accessed on the 14 September 2016), as this database is more complete than CRIBI.

### 4.6. Sample-Specific Transcripts

Venn Diagrams were used to assess the transcripts that were exclusively present in each sample, using the function venn.diagram from package VennDiagram (1.6.17) [60]. The transcripts exclusive to each treatment (RDI: RPKM > 1 and SDI: RPKM ≤ 1) were selected to compare between the two irrigation strategies. The argument scaled = FALSE was applied to avoid proportional circles.

### 4.7. Real Time Quantitative PCR (RT-qPCR)

cDNA was prepared using oligo-dT primers (STABvida, Caparica, Portugal) and the kit RevertAid Reverse Transcriptase (Thermo Fisher Scientific, Waltham, MA, USA), according to the manufacturer’s instructions, using 0.5 µg RNA. The cDNA thus obtained was stored at –20 °C until further analysis.

The transcripts used for qPCR were within functional categories of interest, such as response to stress, hormones, signaling, metabolism, and regulation, and were significantly regulated in the RNAseq analysis. Primers were designed with Beacon designer 4 (PREMIER Biosoft, Davis, CA, USA) Software. The sequences used were retrieved from the RNA-seq experiment, and the levels of expression in that experiment and the primer sequences used for RT-qPCR are indicated in Table S3. RT-qPCR was performed in an iQ5 Real Time PCR (Bio-Rad, Hercules, CA, USA), and 96 wells transparent reaction plates were used. Three biological replicates and two technical replicates were conducted. The mix was composed of EvaGreen Master mix (SsoFast_EvaGreen supermix, Bio-Rad), prepared according to the manufacturer’s instructions, 10 µM of primer, diluted cDNA (2.5 ng µL^−1^), and Mili-Q H_2_O to obtain a final volume of 20 µL. The PCR program was the following: initial denaturation at 95 °C for 2 min; 50 cycles of denaturation at 95 °C, annealing at 60 °C for 30 s and elongation at 72 °C for 45 s, with a reading of fluorescence at the end of each cycle, followed by a final elongation at 72 °C for 5 min.

The baseline subtracted logarithmic amplification plot of the fluorescence signal (ΔRn) was obtained with the readings gathered between cycles 5 and 17. The Rn threshold was set at 50 to obtain Cq values, that were exported to excel, for quantification. The ΔΔCq method [61] was used to quantify relative gene expression, using as references *Actin 2 (ACT), Vitis vinifera translation initiation factor 3 subunit G (TIF),* and *Vitis vinifera translation initiation factor eIF-2B subunit alpha (TIF-GTP)*. These genes were chosen due to their stability under abiotic stress conditions [62].

### 4.8. Statistical Analysis

Significant variations of gene expression in RDI (using SDI as control) were considered if |log2 (gene expression level)| > 2. The same rule was used in the comparison along berry development, with the phenological stage green pea as control. Variations between veraison and maturation in RDI; and between RDI and SDI in each phenological stage were assessed through Student’s t-test (Excel, Microsoft, Albuquerque, NM, USA) for *p*-value < 0.05.

## 5. Conclusions

In conclusion, primary metabolism was the functional category most affected under severe water stress, followed by signaling and transport. Almost all the genes selected to be studied by RT-qPCR experienced significant increases in expression at veraison, the developmental stage that was most affected by water stress, and a pattern that appears variety-specific. At full maturation, as Tempranillo berries responded to water stress with a down-regulation of general metabolism, gene expression decreased to a minimum, with increases of the genes associated with pathways that confer tolerance to stress.

One of those stress response mechanisms is the induction of *HSPs*. In Tempranillo berries, however, they were relevant in the response to stress at veraison, but not at full maturation. The response to water stress at late developmental stages may fall under the regulation of auxins, as the modulation of the expression of an auxin response repression factor, *AUX22D*, by water stress, indicates. Further, *ERF105* may play a role in the regulation of the expression of *WRKY40*, a TF that controls anthocyanin biosynthesis, and that could be responsible for changes in Tempranillo grape quality under severe drought stress. This response suggests an interplay between ethylene signaling pathways and anthocyanin production, which may have consequences in wine quality.

## Figures and Tables

**Table 1:** Detailed analysis of the transcripts up- and down-regulated in the functional categories Signaling and Transcription Factors; according to the results shown in Figure 2.

		Up-Regulated	Down-Regulated
signaling	Cinase	32	13
	GTPase	4	1
	Calcium sensor	8	0
	Fosfatase	4	3
	ABA	4	2
	Auxin	9	2
	Ethylene	5	2
	Cytokinin	4	0
	Jasmonic salicylate	4	1
transcription factors	WRKY	2	0
	ABI3	1	0
	BZIP	3	2
	Zinc Finger	9	5
	MYB	9	1
	NAC	2	0
	GIF	1	0
	RWP-RK	1	0
	Homeobox domain	3	0
	BHLH	1	1
	G2	1	1
	Regulation overview	17	7

**Table 2:** Top ten up- and down-regulated DEGs obtained at full maturation through RNA-seq analysis. An empty gene name entry indicates that no name was found on CRIBI/TAIR.

	Transcript ID	Ratio	RPKM RM	RPKM SM	Gene Name	Gene Functional Category
Down-regulated	VIT_217s0053g00010.1	69	1099	16		No Name
	VIT_209s0018g00240.2	32	32	0	WRKY40	Regulation overview
	VIT_206s0004g03550.1	29	29	0	APX1	Metabolism
	VIT_211s0052g01710.3	27	27	0		Metabolism
	VIT_205s0102g00140.1	25	454	18		No Name
	VIT_218s0001g12240.1	22	22	0	ERF1-3	Metabolism
	VIT_212s0028g01130.1	18	1735	97		No Name
	VIT_210s0003g04505.1	16	524	33		No Name
	VIT_200s0505g00060.1	16	68	4		Metabolism
	VIT_207s0031g00190.1	16	16	0	DEAR3	Signaling
	VIT_217s0053g00010.1	69	1099	16		No Name
Up-regulated	VIT_212s0035g01900.3	−119	0	118	PME44	Cellular process
	VIT_204s0044g00710.5	−16	3	49	UGP2	Metabolism
	VIT_210s0003g04880.1	−15	0	15	RFNR2	Metabolism
	VIT_208s0040g00870.1	−14	0	14	BZIP17	Regulation overview
	VIT_218s0122g01440.3	−12	0	12	AAE1	Unclear
	VIT_200s0338g00020.1	−11	0	11		Unknown
	VIT_205s0077g00430.1	−10	2	17	GolS1	Metabolism
	VIT_211s0052g01720.1	−10	0	10	ARF3	Cellular process
	VIT_202s0087g00770.2	−10	0	10	RLI2	Transport overview

## Data Availability

The data are contained within the article.

## Supplementary Materials

The following supporting information can be downloaded at: https://www.mdpi.com/article/10.3390/plants12091778/s1, Table S1. Sequencing results, in terms of read counts (# reads), fragment length and GC percentage (%GC) before and after trimming, in the two SDI and RDI full maturation samples. Table S2. List of DEGs in each pairwise comparison (RPKM, FC, pvalue, expression index in each sample, and gene description). Table S3. List of Differentially Expressed Genes (DEGs) obtained through RNA-seq and further analyzed through RT-qPCR. The primers utilized in RT-qPCR and the expression ratios obtained in RNA-seq are also presented. Figure S1. Correlation between the logarithmized Reads per Kilobase transcript per Million reads (RPKMs) of two biological replicates (in the X and Y axes). Dots represent the transcripts. Pearson Method was used to calculate linear regression (black line). Coefficient of determination (R-squared) and the linear regression equation are provided; (a) full maturation SDI; (b) full maturation RDI.
