# Supplementary material for: Modulation of the Berry Skin Transcriptome of cv. Tempranillo Induced by Water Stress Levels
Source: Plants (Basel). 2023 Apr 26;12(9):1778. doi: 10.3390/plants12091778 (PMC10180983; doi:10.3390/plants12091778)
Supplement: Supplementary file 1 [file plants-12-01778-s001.zip › Supplementary Figure.pdf]

Supplementary Figure. Correlation between biological replicates in RNA-seq.

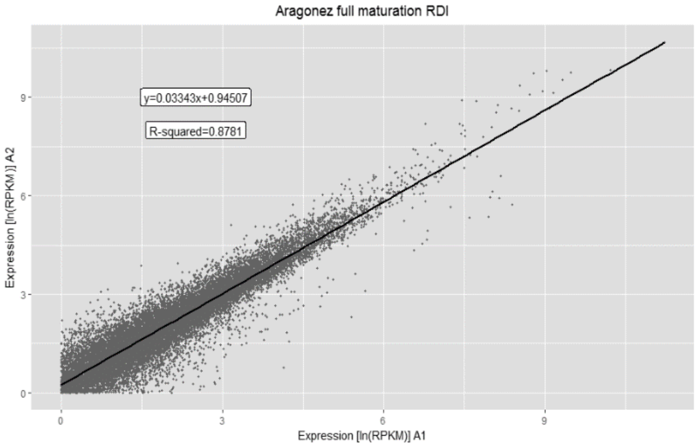

(**a**)

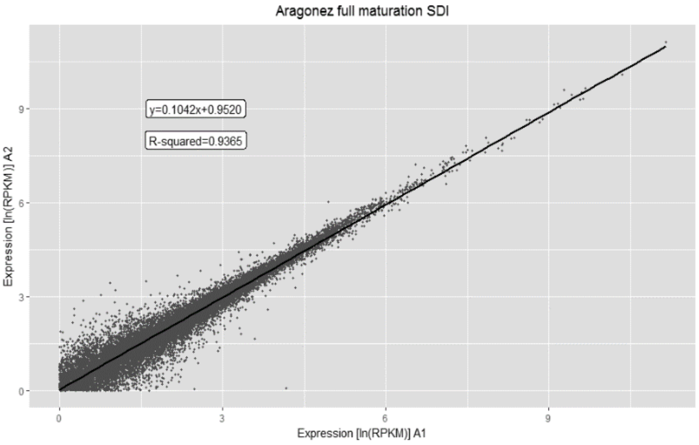

(**b**)

Supplementary Figure S1. Correlation between the logarithmized Reads per Kilobase transcript per Million reads (RPKMs) of two biological replicates (in the X and Y axes). Dots represent the transcripts. Pearson Method was used to calculate linear regression (black line). Coefficient of determination (R-squared) and the linear regression equation are provided; (**a**) full maturation SDI; (**b**) full maturation RDI.
